# Assessment of habitat suitability of the snow leopard (*Panthera uncia*) in Qomolangma National Nature Reserve based on MaxEnt modeling

**DOI:** 10.24272/j.issn.2095-8137.2018.057

**Published:** 2018-05-24

**Authors:** De-Feng Bai, Peng-Ju Chen, Luciano Atzeni, Lhaba Cering, Qian Li, Kun Shi

**Affiliations:** 1 Wildlife Institute, School of Nature Conservation, Beijing Forestry University, Beijing 100083, China; 2 Everest Snow Leopard Conservation Center, Rikaze Xizang 857000, China; 3 Qomolangma National Nature Reserve Administration, Rikaze Xizang 857000, China; 4 Eco-Bridge Continental, Beijing 100085, China

**Keywords:** Qomolangma National Nature Reserve, Snow leopard, MaxEnt, Habitat suitability assessment, Tibet

## Abstract

Habitat evaluation constitutes an important and fundamental step in the management of wildlife populations and conservation policy planning. Geographic information system (GIS) and species presence data provide the means by which such evaluation can be done. Maximum Entropy (MaxEnt) is widely used in habitat suitability modeling due to its power of accuracy and additional descriptive properties. To survey snow leopard populations in Qomolangma (Mt. Everest) National Nature Reserve (QNNR), Xizang (Tibet), China, we pooled 127 pugmarks, 415 scrape marks, and 127 non-invasive identifications of the animal along line transects and recorded 87 occurrences through camera traps from 2014–2017. We adopted the MaxEnt model to generate a map highlighting the extent of suitable snow leopard habitat in QNNR. Results showed that the accuracy of the MaxEnt model was excellent (mean AUC=0.921). Precipitation in the driest quarter, ruggedness, elevation, maximum temperature of the warmest month, and annual mean temperature were the main environmental factors influencing habitat suitability for snow leopards, with contribution rates of 20.0%, 14.4%, 13.3%, 8.7%, and 8.2% respectively. The suitable habitat area extended for 7 001.93 km^2^, representing 22.72% of the whole reserve. The regions bordering Nepal were the main suitable snow leopard habitats and consisted of three separate habitat patches. Our findings revealed that precipitation, temperature conditions, ruggedness, and elevations of around 4 000 m a.s.l. influenced snow leopard preferences at the landscape level in QNNR. We advocate further research and cooperation with Nepal to evaluate habitat connectivity and to explore possible proxies of population isolation among these patches. Furthermore, evaluation of subdivisions within the protection zones of QNNR is necessary to improve conservation strategies and enhance protection.

## INTRODUCTION

Wildlife habitat is defined as the surrounding environment where wild animals can accomplish their life cycle (Cody, 1987; Jiang et al., 2012). Habitats supply resources for population persistence, representing a determining factor for survival and successful reproduction (Wang & Chen, 2004; Yang et al., 2000). The habitat suitability index (HSI) is a measure of the ability for a habitat to sustain a species and is an important indicator for the quality of a given habitat (Jin et al., 2008; Lu et al., 2012; Song et al., 2014). Such evaluation constitutes one of the first steps in wildlife protection and management, offering a scientific rationale for the improvement of conservation policies (Liu et al., 2013).

The snow leopard (*Panthera uncia*) is a feline species distributed over 12 countries in Central Asia. It is estimated that China contains approximately 60% of the potential habitat available to snow leopards, who are reported to reside in the western provinces of Xinjiang, Xizang (Tibet), Qinghai, Gansu, Sichuan, and Inner Mongolia (McCarthy & Chapron, 2003; Riordan & Shi, 2010, 2016). The species is currently classified as Vulnerable by the IUCN (International Union for Conservation of Nature and Natural Resources, 2017) and listed as a Class I protected animal by the China Key List (Riordan & Shi, 2010; Wang, 1998). Snow leopard-oriented research spans a diverse range of areas, including abundance and density (Alexander et al., 2015; Jackson et al., 2006), home range (Johansson et al., 2016), diet (Wang et al., 2014), behavior (Li et al., 2013), genetic diversity (Janečka et al., 2017), climate change impact (Aryal et al., 2014d, 2016), human-snow leopard conflict (Aryal et al., 2014b; Chen et al., 2016), and translocation of prey species (Aryal et al., 2013).

Previous studies on snow leopard habitat use indicate two broad components determining such selection. First, snow leopard occurrence is predicted by several abiotic factors such as terrain (slope and aspect) (Sharma et al., 2015; Wolf & Ale, 2009), elevation (Alexander et al., 2016a; Aryal et al., 2014c), snow cover (Aryal et al., 2014c), distance from rivers (Aryal et al., 2014c), and annual mean temperature (Li et al., 2013). The second component is represented by biotic factors such as prey availability (Alexander et al., 2016b; Aryal et al., 2014a; Xu & Luo, 2010) and human activity (Wolf & Ale, 2009). During the 1990s, slightly more than 100 snow leopards were estimated to inhabit Qomolangma National Nature Reserve (QNNR). Based on three brief surveys, Jackson et al. (1994) estimated that “good” habitat totaled approximately 8 000 km^2^, or about 25% of QNNR’s area. Furthermore, in May–June 2014 and May 2015, a preliminary snow leopard presence survey (Chen et al., 2017) and a human-carnivore conflict survey (Chen et al., 2016) were conducted in QNNR, respectively. A more comprehensive and interdisciplinary study was also conducted to provide an evidential basis for the formulation of effective conservation policies and programs (Chen et al., 2017). Based on the above studies, habitat suitability assessment was deemed necessary for a systematic survey of the snow leopard population in QNNR. Several studies have been conducted to estimate the extent of suitable habitat covering the whole snow leopard range (McCarthy et al., 2016; Hunter & Jackson, 1997; Fox, 1994). However, habitat suitability assessment at the regional level has not yet been reported, resulting in a gap between research and local conservation actions.

Recently, with the development of 3S (GIS, RS, GPS) techniques, multiple models have been used to assess suitable habitat distribution, including mechanism models (Ouyang et al., 2001; Xu et al., 2006), regression models (Schadt et al., 2002), and ecological niche models (Su et al., 2015; Xu & Luo, 2010; Wang et al., 2008; Phillips et al., 2006). The maximum entropy (MaxEnt) model is an ecological niche model, which has been increasingly used to assess wildlife habitat distribution (Clements et al., 2012; Wilting et al., 2010). MaxEnt models were originally formulated to estimate the presence density of a target species across a landscape (Phillips et al., 2006), but have since been applied to model species distribution and environmental niches, relying on “presence-only” data and environmental predictors, thereby avoiding bias and leading to accurate results (Merow et al., 2013; Pearson et al., 2007; Phillips et al., 2006). MaxEnt models are reliable, straightforward, and allow data to be easily obtained (Merow & Silander, 2014; Phillips & Dudík, 2008; Radosavljevic & Anderson, 2014). These models are suitable for the prediction of species habitat distribution at the whole reserve level, and an appropriate model for habitat suitability analysis (Haegeman & Etienne, 2010; Stachura-Skierczyńska et al., 2009; Xing & Hao, 2011).

Understanding the distribution of snow leopard habitat in QNNR is important for the improvement of research outcomes and conservation plans. Thus, our study aimed to: (1) assess the potential habitat distribution for snow leopard in QNNR, along with biotic and abiotic factors of influence using the MaxEnt model; and (2) identify critical areas for snow leopard conservation under the existing functional zones of QNNR.

## MATERIALS AND METHODS

### Study area

Located in the southwest Xizang (Tibet) Autonomous Region, China, QNNR (N27${}^{\circ }$48${}^{\prime }$–N29${}^{\circ }$19${}^{\prime }$, E84${}^{\circ }$27${}^{\prime }$–E88${}^{\circ }$23${}^{\prime }$) was established in 1989 to protect wildlife and ecosystems along the border of China and Nepal. The reserve covers an area of 33 814 km^2^, centering on the world’s highest peak, Mt. Everest. Altitude ranges from 1 440 m to 8 844 m a.s.l.. The average annual temperature is 2.1 ${}^{\circ }$C and total annual rainfall is 270.5 mm. Some 81 species of mammals, 342 birds, 29 amphibians and reptiles, and 8 fish inhabit the reserve (QNNR Administration, unpublished). Large mammals include the snow leopard (*Panthera uncia*), wolf (*Canis lupus*), lynx (*Lynx lynx*), brown bear (*Ursus arctos*), leopard (*Panthera pardus*), blue sheep (*Pseudois nayaur*), wild ass (*Equus kiang*), Tibetan gazelle (*Procapra piticaudata*), and Himalayan tahr (*Hemitragus jemlahicus*) (Chen et al., 2017). QNNR consists mainly of two broad ecosystems: that is, semi-humid mountain forest and semi-arid shrub along the southern and northern parts, respectively. Within these, vegetation varies across different sub-ecosystems. As altitude increases, 10 distinct sub-ecosystems can be observed progressively, including subtropical evergreen broad-leaved forest, subtropical semi-green broad-leaved forest, subtropical evergreen coniferous forest, warm temperature green coniferous forest, warm temperature sclerophyllous evergreen broad-leaved forest, subalpine temperature zone evergreen coniferous forest, subalpine temperature zone broad-leaved deciduous forest, alpine sub-frigid zone shrubland and grassland, alpine sub-frigid zone periglacial and alpine snow zone (QNNR Administration, unpublished). The perpendicular width of each vertical ecological system ranges from hundreds to thousands of meters, and each is very sensitive to external environment change (QNNR Administration, unpublished). For the different functional zones in QNNR, any disturbance is restricted in the core zone, scientific research can be conducted in the buffer area, and some human activity can occur in the experimental zone.

### Model selection

Information science is the basis of MaxEnt models, which are widely used in many academic disciplines, as proposed by Jaynes (1957a, 1957b) and more recent studies (Jaynes & Bretthorst, 2003; Xing & Hao, 2011). The MaxEnt model is based upon ecological niche theory, relying on information from species “presence” data to explore the possible distribution of a target species within a study area. In 2004, MaxEnt software was developed to assess and evaluate suitable habitat for target species with good predictive power (Phillips et al., 2006). Occupancy models and generalized linear models (GLM) are also used to explore the relationship between species habitat selection and environmental factors (Alexander et al., 2015; MacKenzie et al., 2006; Wang et al., 2018). However, GLM and occupancy models exhibit similar limitations, such as the need for presence/absence or detection/non-detection data, which can introduce difficulties when an expansive study area is needed to detect rare species like snow leopards (Alexander et al., 2016a; MacKenzie et al., 2006). The MaxEnt model provides a self-checking function with automatically generated receiver operating characteristic (ROC) curves. Furthermore, to understand the regional level habitat distribution of snow leopards in QNNR, presence records are more likely to be accessible, and thus the MaxEnt model has an advantage over other types of models (Su et al., 2015; Liu et al., 2013). Therefore, the MaxEnt model was selected in this study to explore the potential suitable habitat of snow leopards in QNNR.

### Snow leopard “presence” data

**Figure 1:**
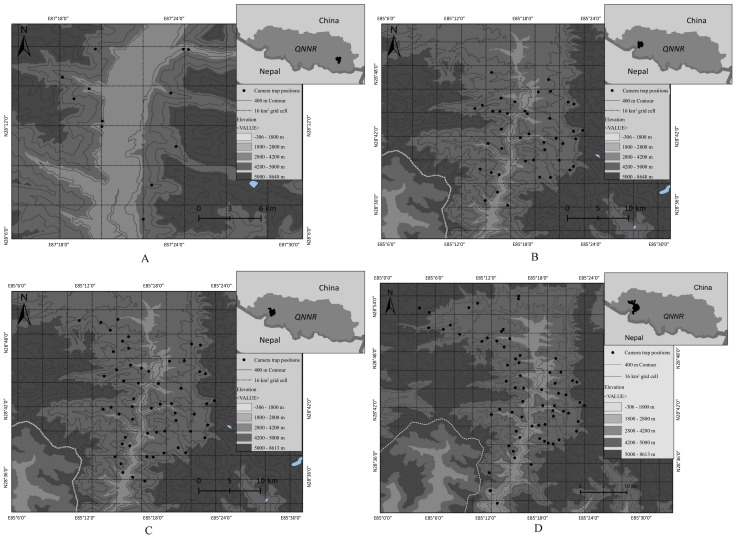
Location of camera traps for snow leopard surveys in Qomolangma National Nature Reserve, Xizang (Tibet)

In 2014, a preliminary survey was conducted in four areas with 33 camera traps around the villages of Zhalong (Jilong County), Dacang (Dingjie County), Riwu and Qudang (Dingri County) (Chen et al., 2017). Other study areas were later selected to estimate snow leopard abundance and density with high intensity. Our study area was divided into several 4 km×4 km grids. We selected sampling transects and camera locations according to our knowledge of the ecological requirements of snow leopards and the experience of our local guide to maximize the detectability of animals in each grid. In 2015, following the preliminary survey, we selected Zhalong (Jilong County) and Qudang (Dingri County) as study areas. In total, 83 and 23 camera traps (Ltl Acorn) were systematically set up over 400 km^2^ in the Zhalong study area and 208 km^2^ in the Qudang study area between October and November 2015, respectively. In summer 2016 (April to May), 112 camera traps (Ltl Acorn) were set up over 480 km^2^ in the Zhalong study area. In winter 2016, an additional 68 camera trap sites were set up over 790 km^2^ in another part of the Jilong study area. In winter 2017, a snow leopard survey was conducted over 750 km^2^ in the Zhaxizong study area (Dingri County). We recorded snow leopard signs (scats, tracks, scent marks, scrapes, bedding, and hair) along the most likely routes (ridgelines, hillsides, prey resource-rich places, and higher rugged places). Camera stations were placed with a minimum spacing of 1 km to maximize the number of individuals caught and adequately recapture individuals at different camera traps (Alexander et al., 2015). We placed two cameras in each site to capture both sides of the animal (Jackson et al., 2006). Within each grid, two or more camera stations were established. The Zhalong study area (Jilong County) was expanded from 400 km^2^ in 2015 to 790 km^2^ in 2016. The transects used in 2015 were fixed for later surveys, but camera trap sites were not (Figure 1).

A: Qudang Study Area, Dingri County, winter 2015. B: Zhalong Study Area, Jilong County, winter 2015. C: Zhalong Study Area, Jilong County, summer 2016. D: Zhalong Study Area, Jilong County, winter 2016.

For non-invasive genetics, we collected samples that belonged to snow leopards along transects to set up camera traps. The selection of feces was based upon shape, hair content, and terrain type or was found in association with other marking signs particular to this species. We collected scat samples using silica gel drying agent (Boitani & Powell, 2012; Wasser et al., 1997). In the field, tubes containing silica desiccant were stored away from light in a dry and cool environment and were immediately stored at −20 ${}^{\circ }$C after arrival at the laboratory. DNA from scat samples was extracted using a QIAamp DNA Stool Mini Kit (Qiagen, USA) following a modified extraction protocol under a dedicated UV-light laminar flow cabinet, physically separated by the pre-PCR area. Each batch of extracted samples was processed along with a negative control to account for possible contamination, with results screened on 1% agarose gel. Species identification was initially conducted through amplification of a 110 base pairs-portion of the cyt *b* (cyt *b*) gene using carnivore-specific primers, as described in Farrell et al. (2000) (forward: 5${}^{\prime }$-TATTCTTTATCTGCCTATACATACACG-3${}^{\prime }$; reverse: 5${}^{\prime }$-AAACTGCAGCCCCTCAGAATGATATTTGTCCTCA-3${}^{\prime }$). PCR analysis was conducted under a dedicated UV-sterilized laminar flow hood using Premix Taq (Ex Taq Version 2.0; Takara Biotechnology) to a final volume of 20 μL containing 0.2 mmol/L of each dNTP, 2 mmol/L of Mg^2+^, 0.4 μmol/L of each primer, 7.4 μL of DNAase/RNAase-free ultrapure water (Tiangen Biotech, Beijing), and 1 μL of extracted DNA. PCR conditions consisted of an initial denaturation step of 10 min at 94 ${}^{\circ }$C, 35 cycles at 94 ${}^{\circ }$C for 30 s, 57 ${}^{\circ }$C for 45 s, and 72 ${}^{\circ }$C for 45 s, followed by a final elongation step at 72 ${}^{\circ }$C for 10 min. All amplifications included one positive snow leopard reference sample and a blank control to account for contamination. Results were screened by 1% agarose gel electrophoresis. Successful PCR products were sequenced through an Applied Biosystems ABI 3730XL system by SinoGenoMax Limited Company (Beijing, China). Readings were assembled in Geneious 10.1 (Biomatters Ltd.) and matched against the NCBI database using BLAST to obtain the percentage of similarity. Only completely assembled sequences were considered for the dataset. Samples yielding incomplete assemblies, along with unsuccessful PCRs for cyt *b* and non-carnivore species identifications, were re-screened targeting a 126 base pairs-portion of the *ATP6* gene using the carnivore-specific primers described in Chaves et al. (2012) (DF3: 5${}^{\prime }$-AACGAAAATCTATTCGCCTCT-3${}^{\prime }$; DR1: 5${}^{\prime }$-CCAGTATTTGTTTTGATGTTAGTTG-3${}^{\prime }$). The primers described in Farrell et al. (2000) were used to target prey species DNA (Chaves et al., 2012). PCR volumes were identical to the protocol used for cyt *b*, as were the PCR conditions, except for the annealing temperature (51 ${}^{\circ }$C for 45 s).

Feces, scent marks, and killings could be attributed to carnivores other than snow leopards; however, pugmarks and scrapes are easily recognized. Therefore, pugmarks, scrapes, and feces identified as belonging to snow leopards were selected to create a suitable habitat distribution simulation in QNNR (Jackson & Hunter, 1996; Janečka et al., 2008). We used 1-km^2^ grid cells for the MaxEnt niche-based modeling (Phillips et al., 2006). One snow leopard presence per cell was used and repeated location data were removed. A total of 222 GPS snow leopard presence points were included for further analysis. However, due to the higher number of field studies in the Jilong area, records were heavily geographically unbalanced, with the Jilong area containing more than 50% of all records. We thus further reduced the number of records in the Jilong area by randomly selecting records to produce the same survey effort as outside the Jilong area. As only 59 records were included for analysis outside the Jilong area (east QNNR), we only included 59 records from the Jilong area (west QNNR).

### Environment variables

We tested the relationship of snow leopard presence data with possible influencing factors. We identified two main categories related to suitable habitat distribution, namely abiotic factors (elevation, slope, aspect, ruggedness, land use type, and distance factors) and bioclimatic and biotic factors (prey species, human related factors) (Alexander et al., 2016a, 2016b; Sharma et al., 2015). We selected elevation, slope, aspect, ruggedness, land use type, and bioclimatic factors for landscape level analysis. We used snow leopard presence points and extracted 19 bioclimatic variables of our study area from WorldClim (www.worldclim.org) data (Table 1). In ArcGIS, elevation, aspect, slope, and ruggedness were determined using a digital elevation model (DEM) layer, with all variables clipped to our study areas. Ruggedness was extracted based on the method of Riley et al. (1999). The Topographic Ruggedness Index (TRI) was used to express the elevation difference between adjacent cells (all eight first-order neighbors within a quadratic grid) of a 90-m-resolution DEM on a scale from 1 (level) to 7 (extremely rugged). Land use and land cover were extracted from “GLOBCOVER 2009” obtained from the University of Louvain (UCLouvain) and European Space Agency (ESA). Distance factors (distances to roads, settlements, and rivers) and biotic factors were excluded due to the small-scale snow leopard and prey species presence data. We then extracted the values of each variable corresponding to the presence locations of snow leopards to perform correlation analyses (Supplementary Table S1). After removing 10 highly correlated variables (>0.85), we used the remaining 14 variables for final analysis.

**Table 1:** Variables used for modeling

SN	Variable	Description	Source
1	BIO1 (Annual mean temperature)	Continuous	http://www.worldclim.org/bioclim (Hijmans et al., 2005) (1–19)
2	BIO2 (Mean diurnal range)	Continuous		
3	BIO3 (Isothermality)	Continuous		
4	BIO4 (Temperature seasonality)	Continuous		
5	BIO5 (Max temperature of warmest month)	Continuous		
6	BIO6 (Min temperature of coldest month)	Continuous		
7	BIO7 (Temperature annual range)	Continuous		
8	BIO8 (Mean temperature of wettest quarter	Continuous		
9	BIO9 (Mean temperature of driest quarter)	Continuous		
10	BIO10 (Mean temperature of warmest quarter)	Continuous		
11	BIO11 (Mean temperature of coldest quarter)	Continuous		
12	BIO12 (Annual precipitation)	Continuous		
13	BIO13 (Precipitation of wettest month)	Continuous		
14	BIO14 (Precipitation of driest month)	Continuous		
15	BIO15 (Precipitation seasonality) (Coefficient of variation)	Continuous		
16	BIO16 (Precipitation of wettest quarter)	Continuous		
17	BIO17 (Precipitation of driest quarter)	Continuous		
18	BIO18 (Precipitation of warmest quarter)	Continuous		
19	BIO19 (Precipitation of coldest quarter)	Continuous		
20	Land cover	Categorical (Irrigated croplands, Rainfed croplands, Mosaic Croplands/Vegetation, Mosaic Vegetation/Croplands, Closed to open broadleaved evergreen or semi-deciduous forest, Closed broadleaved deciduous forest, Closed needle-leaved evergreen forest, Closed to open mixed broadleaved and needle-leaved forest, Mosaic Forest-Shrubland/Grassland, Mosaic Grassland, Forest-Shrubland, Closed to open shrubland, Closed to open grassland, Sparse vegetation, Close to open vegetation regularly flooded, Bare areas, Water bodies, Permanent snow and ice)	GLOBCOVER 2009 form University of Louvain (UCLouvain) and European Space Agency (ESA) (20)
21	Aspect	Categorical (Flat; North: 0${}^{\circ }$–22.5${}^{\circ }$; Northeast: 22.5${}^{\circ }$–67.5${}^{\circ }$; East: 67.5${}^{\circ }$–112.5${}^{\circ }$; Southeast: 112.5${}^{\circ }$–157.5${}^{\circ }$; South: 157.5${}^{\circ }$–202.5${}^{\circ }$; Southwest: 202.5${}^{\circ }$–247.5${}^{\circ }$; West: 247.5${}^{\circ }$–292.5${}^{\circ }$; Northwest: 292.5${}^{\circ }$–337.5${}^{\circ }$; North: 337.5${}^{\circ }$–360${}^{\circ }$)	GMTED2010 (21–24)
22	Slope	Categorical (0–4.375, 4.375–9.310, 9.310–14.216, 14.216–19.223, 19.223–24.395, 24.395–29.823, 29.823–35.773, 35.773–43.812, 43.812–72.092)		
23	Elevation	Continuous (m)		
24	Ruggedness	Continuous		

SN: Serial number.

### Simulation procedure

Snow leopard presence data and selected variables were adapted to the format required for MaxEnt software (v 3.3.3k) (Phillips et al., 2006). We selected 75% of snow leopard presence data to build the model, with the remaining 25% used for model verification. We included 10 replicates in our analysis. We used a jackknife estimator to detect the importance of each variable. Sensitivity analysis was done for each variable with logistic output format. Results of the MaxEnt model were verified by ROC values: that is, rejected with a ROC value 0.5–0.6; poor with 0.6–0.7; normal with 0.7–0.8; good with 0.8–0.9; and excellent with 0.9–1.0. According to the expert experience method (Swets, 1988), the output results were used in ArcGIS 10.2 to reclassify the suitable snow leopard habitat distribution map. Using the MaxEnt model to evaluate habitat suitability in QNNR, the ASCII format file was imported into ArcGIS 10.2 for transformation into floating raster data. The floating raster was reclassified into low grade habitat (0–0.14), moderately suitable habitat (0.14–0.42), and highly suitable habitat (0.42–1). Focal statistics in ArcGIS were used to smooth the raster map to obtain the suitable snow leopard habitat distribution map in QNNR.

## RESULTS

### Snow leopard presence data records

In 2014, we recorded five snow leopard pugmark sites and 65 scrape sites, and 17 camera trap sites captured snow leopard photographs. In winter 2015, we recorded 38 pugmark sites, 111 scrape sites, and 27 camera trap sites with photographs. In summer 2016, we recorded 35 pugmark sites, 131 scrape sites, and 44 camera trap sites with photographs. In winter 2016, we recorded 39 pugmark sites, 73 scrape sites, and 32 camera trap sites with photographs. In winter 2017, we recorded 10 pugmark sites and 35 scrape sites.

We collected a total of 52, 84, 135, and 72 fecal samples in summer 2014, winter 2015, summer 2016, and winter 2016, respectively. In 2014, we positively identified 29 samples through cyt *b*, 21 of which belonged to snow leopards. The 23 unsuccessful samples, including two incomplete assemblies, produced 15 snow leopard identifications through the *ATP6* gene. In total, we identified 84% of samples (44), with snow leopard identifications accounting for 69% of the total (36). Of the 86 samples collected in winter 2015, 43 were positively identified as a carnivore species using cyt *b*, of which 14 samples belonged to snow leopard. The remaining 43 were screened using the *ATP6* gene; however, out of the positive PCRs, identification was only possible for one sample, which belonged to a snow leopard. In total, for samples collected in 2015, we identified 44 samples (51%), 15 of which were snow leopards (17% of total). Species identification of the 135 samples collected in spring 2016 yielded 71 assembled predator species identifications, 23 of which were snow leopards. Out of the 64 samples selected for a second amplification, 55 failed to amplify, five belonged to blue sheep (*Pseudois nayaur*), one to yak (*Bos grunniens*), and three were partial assemblies belonging to a carnivore species (either forward or reverse strand). We then amplified 64 samples for the *ATP6* gene, yielding 31 positive carnivore identifications, with 26 belonging to snow leopards. In summary, 102 samples (75.5%) were successfully identified, with snow leopards representing 36.2% of the total (49 identifications). For winter 2016, we identified 24 carnivores out of 72 samples through cyt *b*, 11 of which were identified as snow leopard. The remaining 48, amplified through the *ATP6* gene, yielded 19 carnivores, with snow leopards identified 16 times. In total, we identified 43 carnivores in 43 occasions (60% of total), and snow leopards represented 37.5% of total fecal samples. Pooling all samples together over the four field seasons, we collected 345 fecal samples and identified 233 (66%) as belonging to a predator species. Snow leopards were identified 127 times, representing 36.8% of the total collected scats and 54.5% of the successful identifications. Results are summarized in Table 2.

**Table 2:** Identification results from molecular scatology

Field Season	Scats	Cyt *b*+	*ATP6*+	SLs	Failed
Summer 2014	52	29	15	36	8
Winter 2015	84	43	1	15	42
Summer 2016	135	71	31	49	33
Winter 2016	72	24	19	27	29
Total	343	167	66	127	112

Cyt *b*+ and *ATP6*+ indicate success of identification using the two markers. SLs: Snow leopards.

**Figure 2:**
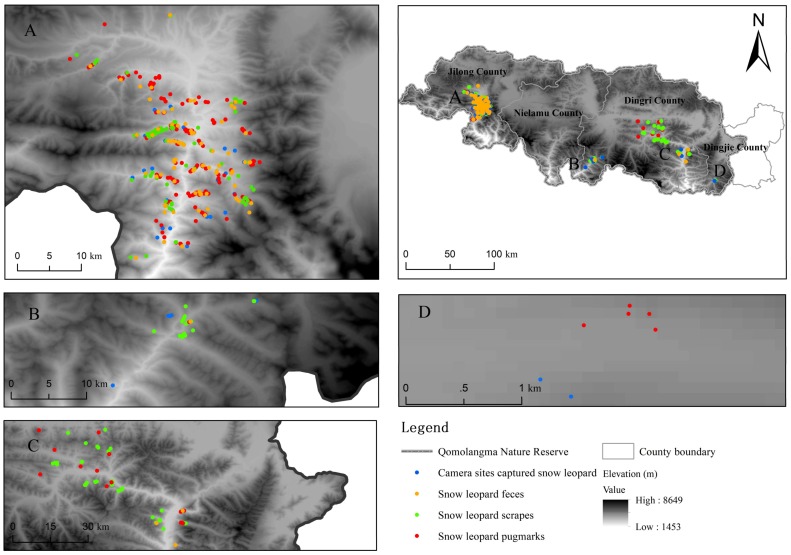
Snow leopard presence data collected in QNNR

In total, 127 pugmark sites, 415 scrape sites, and 127 molecular identifications of snow leopard were recorded. In addition, 120 camera trap sites captured snow leopard images (Figure 2).

### MaxEnt prediction evaluation

**Figure 3:**
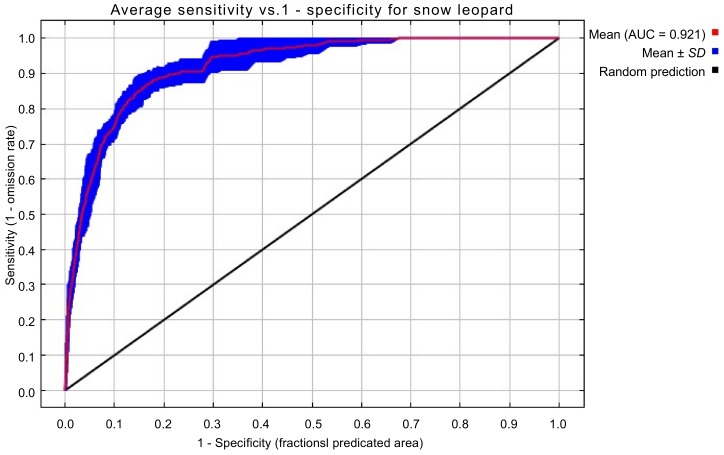
ROC verification of distribution of suitable snow leopard habitat in QNNR

The ROC results (Figure 3) showed a mean AUC value of 0.921, indicating that the predictions obtained from the MaxEnt model were excellent.

### Suitable snow leopard habitat distribution with environment factors

According to the suitable habitat distribution map, we determined that suitable habitat was located mainly along mountain areas near the Nepalese border. We identified three distinct separated patches: (1) Zhalong and Gongdan areas in Jilong county; (2) Chabuling area in Nielamu county and Rongxia area in Dingri county; and, (3) Qudang area and perimeter zone in Dingri county.

**Figure 4:**
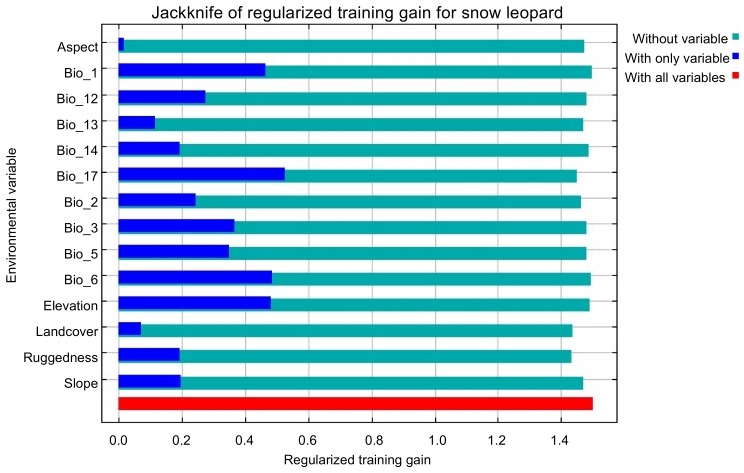
Jackknife test of environmental variables in training data by MaxEnt

The jackknife estimator results (Figure 4; Table 3) showed that precipitation in the driest quarter (BIO17; 20.0%), ruggedness (14.4%), elevation (13.3%), maximum temperature of the warmest month (BIO5; 8.7%), and annual mean temperature (BIO1; 8.2%) were the main factors contributing to snow leopard habitat selection. The importance rates in the MaxEnt model prediction indicated that ruggedness (34.4%), mean diurnal range (BIO2; 16.9%), maximum temperature of the warmest month (BIO5; 11.3%), annual precipitation (BIO12; 8.8%), and precipitation in the driest quarter (BIO17; 7.9%) were the five main factors affecting snow leopard habitat preferences (Table 3; Table 4).

**Table 3:** Contribution and permutation importance values of environmental variables

Environmental variables	Contribution (%)	Permutation importance (%)
Aspect	1.3141	1.38
BIO1	8.1786	0.4244
BIO12	6.8994	8.8319
BIO13	4.214	2.6905
BIO14	0.7669	2.2132
BIO17	19.9978	7.8506
BIO2	6.3171	16.8578
BIO3	4.4267	3.8942
BIO5	8.7044	11.2613
BIO6	4.1076	0.508
Elevation	13.3151	1.6187
Land cover	2.3265	3.9645
Ruggedness	14.3777	34.3892
Slope	5.154	4.1157

**Table 4:** Cumulative and logistic thresholds and corresponding omission rates used for modeling

Cumulative threshold	Logistic threshold	Description	Fractional predicted area	Training omission rate
1.000	0.015	Fixed cumulative value 1	0.641	0.000
5.000	0.056	Fixed cumulative value 5	0.382	0.014
10.000	0.113	Fixed cumulative value 10	0.262	0.047
3.590	0.042	Minimum training presence	0.464	0.000
18.200	0.203	10 percentile training presence	0.173	0.099
24.048	0.261	Equal training sensitivity and specificity	0.128	0.128
30.946	0.330	Maximum training sensitivity plus specificity	0.096	0.133
5.371	0.060	Balance training omission predicted area and threshold value	0.376	0.006
13.893	0.159	Equate entropy of thresholded and original distributions	0.210	0.086

**Figure 5:**
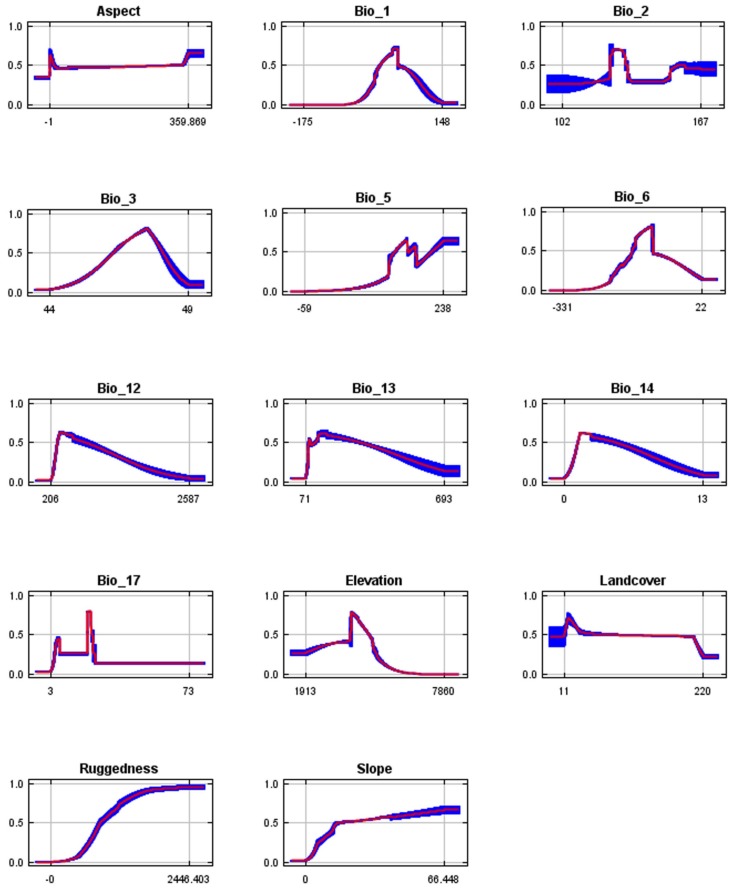
Response curve of selected variables for snow leopard habitat suitability in QNNR

Sensitivity analysis determined the influence of each factor on snow leopard habitat distribution (Figure 5). The altitude in areas suitable for snow leopard habitat was between 3 900 m and 5 000 m a.s.l.. The ruggedness range most suitable for snow leopards was between 1 000 m and 1 300 m a.s.l. where the terrain was extremely rugged. Consequently, snow leopards preferred to use more rugged terrain at elevations of around 4 000 m a.s.l..

### Distribution of suitable snow leopard habitat in QNNR

**Figure 6:**
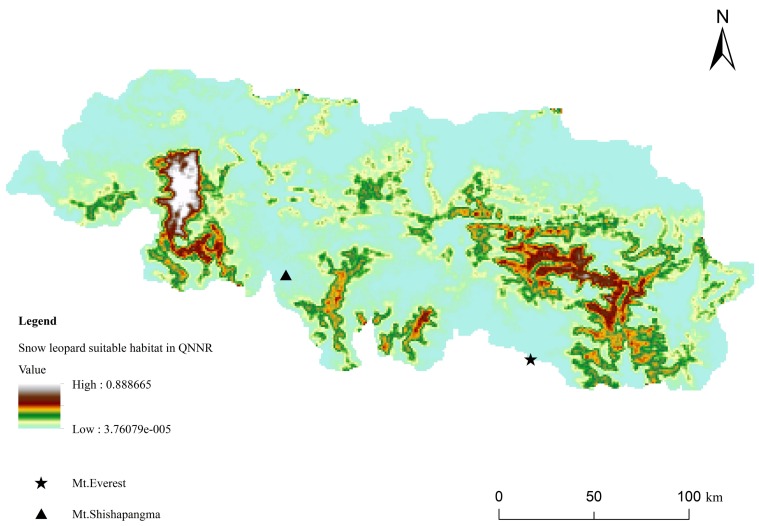
Distribution of suitable snow leopard habitat in QNNR

**Figure 7:**
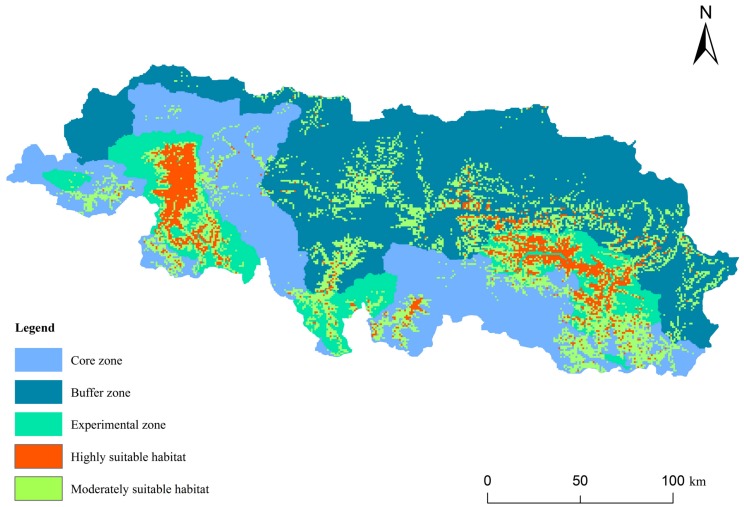
Distribution of suitable snow leopard habitat in different functional zones in QNNR

From the reclassified map, the area of each habitat class (low grade habitat, moderately suitable habitat, and highly suitable habitat) was calculated. The area of highly suitable habitat in QNNR was 1 756.55 km^2^, moderately suitable habitat was 5 245.38 km^2^, and low-grade habitat was 23 814.05 km^2^. Thus, the area of relatively good snow leopard habitat totaled 7 001.93 km^2^ in QNNR, accounting for 22.72% of the overall area of QNNR (Figure 6). According to the existing functional zones (core, experimental, and buffer zones) of QNNR (Figure 7), only 23.24% (1 627.52 km^2^/7 001.93 km^2^) of good habitat lies in the core zone, 36.06% (2 525.22 km^2^/7 001.93 km^2^) lies in the buffer zone, and 40.52% (2 837.40 km^2^/7 001.93 km^2^) lies in the experimental zone.

## DISCUSSION

### Environmental factors influencing snow leopard habitat selection

Our results showed that precipitation in the driest quarter (BIO17), ruggedness, elevation, maximum temperature of the warmest month (BIO5), and annual mean temperature (BIO1) were the five main factors influencing snow leopard habitat suitability in QNNR. Wolf & Ale (2009) conducted research in the Sagarmatha National Park (area of 1 148 km^2^) of Nepal and reported that terrain and human activity were the main factors determining snow leopard spatial distribution, whilst prey species had a moderate effect. In Nepal’s Annapurna Conservation Area, Aryal et al. (2014c) indicated that cliffs, grassland, and shrubland at high elevations (3 000–5 000 m a.s.l.) were preferred habitats of snow leopards in the study area (about 2 025 km^2^). A study conducted in Mongolia reported that distribution of prey resources and rugged terrain largely explained changes in snow leopard habitat use (McCarthy et al., 2005). In India, investigation in intensively grazed areas showed that snow leopard habitat-use mainly relied on wild prey species density (Sharma et al., 2015). In China, very little research has been conducted on snow leopard habitat use. A winter habitat-use survey of snow leopards in Tomur National Nature Reserve highlighted that prey resources and principal geographic features (ruggedness, bases of cliffs, and stream beds) were the main factors influencing snow leopard habitat use within the 3 000 km^2^ study area (Xu et al., 2012). In Sanjiangyuan National Nature Reserve, a landscape level analysis of snow leopard habitat using the MaxEnt model indicted that annual average temperature and ruggedness were the two main factors influencing habitat selection (Li et al., 2013). From the above studies, we can conclude that the determining factors of snow leopard habitat selection may differ in different areas. Thus, local surveys on snow leopard habitat selection are critical to adapt conservation needs according to the local context.

Little has been done to understand regional level snow leopard habitat suitability. Our results found that precipitation and temperature conditions at the regional level had a strong influence on suitable snow leopard habitat. Thus, it is likely that climate change will influence snow leopard habitat selection (Li et al., 2016). Among abiotic factors, elevation and ruggedness had a greater influence on habitat suitability. Our study area had the highest average elevation among the whole global range of snow leopards, ranging from 3 500 m to 5 500 m a.s.l.. Our results indicated that snow leopards preferred elevations of around 4 000 m a.s.l. near Mt. Everest. Like previous research, our findings confirmed that snow leopards favored highly to extremely rugged areas, as based on the highest permutation importance values (Li, 2013). Highly rugged areas usually contain large-sized rugged rocks, which may provide shelter for snow leopards, and thus represent an important feature for their survival. The response curves of land cover and slope showed that snow leopards preferred bare and relatively high slope areas. Aspect had little influence on snow leopard habitat suitability in QNNR.

We selected the MaxEnt model as the most appropriate method to accomplish our research aims. However, we recognize that bias may still exist due to the relatively small study area and the inclusion of a limited number of habitat types, mainly due to resource availability and accessibility. This highlights the common practice of research groups to focus economic efforts toward survey areas where the probabilities of encountering a target species are higher. One fundamental assumption of the MaxEnt model is that the entire area of interest has been systematically or randomly sampled (Phillips et al., 2009; Royle et al., 2012). Our survey showed a strong sampling bias toward some regions or environmental features. To account for this factor, we selected 222 presence sites according to the pixel cells of environmental variables. Many occurrence records were available for this study, therefore we adopted spatial filtering and balancing of occurrence data to minimize omission errors (false negatives) and commission errors (false positives) (Kramer-Schadt et al., 2013). Spatial clumping of records was reduced in datasets for MaxEnt model calibration. Environmental variables were tested regarding their autocorrelation relationship. The manipulation of background datasets was considered inaccurate, as it might increase the risk of omission errors for species like snow leopards with a generalist response in many predictors.

Based on the collected data, we primarily selected non-biotic factors for further analysis. However, the limited number of examined factors may be inappropriate (at least to some extent) for our landscape level analysis in QNNR. An increase in the number of evaluated factors is preferable for landscape level investigation, as advocated by Robinson & Weckworth (2016). More meaningful variables (e.g., human-related factors) influencing snow leopard habitat distribution should be included in future research. Understanding snow leopard habitat in QNNR is important, thus this study provides a basis for more in-depth analysis on population densities and represents a starting point to implement wider conservation-oriented studies.

## Suitable habitat distribution

The MaxEnt model was used to assess habitat suitability in QNNR and, according to the AUC values, results were excellent. We showed that suitable snow leopard habitat in QNNR was mainly located along the border with Nepal, with three distinct habitat patches detected within the nature reserve (Figure 6). This habitat separation might be due to the presence of very high mountains, including Mt. Everest and the Xixiabangma area (Figure 6). According to the geographical features and local knowledge (personal communication), snow leopards are reported to exist along the southern border of the Xixiabangma area and in the northern area of Mt. Everest (near base camp). Using the HIS method, Jackson et al. (1994) described preliminary results for suitable snow leopard habitat, showing such habitat to be mainly located in the western area of QNNR. Our results indicated that “good” snow leopard habitat area was about 1 000 km^2^ smaller than the results given by Jackson et al. (1994). This might be a consequence of the increase in human population and activities like tourism (Chen et al., 2017), or because of the variables and methods used for habitat suitability estimation.

Further study should focus on the three unconnected habitat patches to assess whether individual snow leopards are subject to isolation. Cross-boundary cooperative research with Nepal is also necessary. In QNNR, low-grade snow leopard habitat accounted for about 84% of all potential snow leopard habitat and included very flat areas with little shelter or usable rock cover. However, it is necessary to survey these areas to confirm the “absence” of snow leopards and understand natural prey population statuses. Low-grade snow leopard habitat is important for their survival because it constitutes a part of the ecosystem where snow leopards could exist.

Good snow leopard habitat in QNNR mainly lies in the experimental zone, which is not optimal for protection. Therefore, assessing the efficiency of the functional zones in QNNR is needed in order to implement better conservation strategies and promote new protection policies.

QNNR is located in the core area of the Himalayas and is an important area for global snow leopard conservation (McCarthy & Chapron, 2003). Since its establishment 30 years ago, no-grazing and controlled-grazing policies have been implemented in QNNR; however, the contradiction between protection and development is still prominent (Chen et al., 2016). To improve protection of rare species and ecosystems in QNNR, it is imperative to strengthen management of the whole nature reserve, accounting for target species occurrence and human conflict.

## Supplementary Material

http://www.zoores.ac.cn/fileup/2095-8137/SUPPL/20180803181728.zip
